# Procedural separation of appetitive and consummatory behaviors in operant ethanol self‐administration: A review and open‐source analytical framework

**DOI:** 10.1111/acer.70237

**Published:** 2026-02-03

**Authors:** Olivia A. Colarusso, Jeffrey L. Weiner

**Affiliations:** ^1^ Department of Translational Neuroscience Wake Forest University School of Medicine Winston‐Salem North Carolina USA

**Keywords:** appetitive behaviors, consummatory behaviors, lickometry, operant self‐administration, sipper model

## Abstract

Despite decades of research, no new FDA‐approved medications for alcohol use disorder (AUD) have emerged in over 25 years. Enhancing the translational relevance of preclinical models by more precisely capturing the behavioral and neurobiological features of AUD offers a promising path toward identifying novel therapeutic targets. Operant self‐administration paradigms are essential for modeling voluntary ethanol intake in rodents, yet traditional approaches often confound appetitive (seeking) and consummatory (intake) behaviors. A biphasic sipper model developed by Hank Samson's laboratory addressed this limitation by allowing extended, uninterrupted access to ethanol following operant responding, enabling a clearer dissociation between seeking and consumption. In this review, we synthesize key findings from studies employing this methodology which investigated the behavioral and neurobiological mechanisms underlying alcohol use. We emphasize how over two decades of research employing this approach have demonstrated that ethanol‐directed behaviors are dynamic processes, shaped by internal states, environmental cues, and prior experience. Finally, we introduce an open‐source analytical framework in the R programming language designed to standardize the analysis of high‐resolution temporal data generated by the biphasic sipper paradigm. Together, these methodological and analytical advances enhance the translational potential of preclinical models and may ultimately aid in the discovery of novel therapeutic targets for AUD.

## Graphical abstract

This review highlights the biphasic sipper model pioneered by Dr. Hank Samson's laboratory, which separates appetitive and consummatory behaviors in rodent ethanol self‐administration. Over two decades of research using this paradigm reveals that alcohol‐directed behaviors are dynamic, shaped by internal states, cues, and experience. We also introduce an open‐source R framework for analyzing high‐resolution temporal data. Together, these advances strengthen preclinical models' ability to inform clinical interventions and may accelerate discovery of novel therapeutic targets for alcohol use disorder.

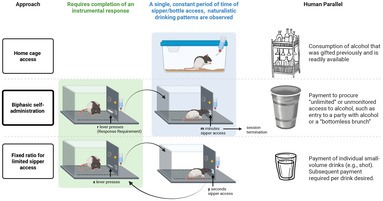

## INTRODUCTION

Studying alcohol has been of great interest for decades due to the growing understanding of alcohol's deleterious effects on health and society. Recent statistics indicate that 62% of Americans aged 12 and older consumed alcohol in the past year and 29.5 million individuals (10%) were diagnosed with alcohol use disorder (AUD) (National Survey on Drug Use and Health, 2023). Moreover, alcohol accounted for 3% of all deaths in America during 2020 (99,000 lives) (White et al., 2022). Core symptoms of AUD, as outlined in the 5th edition of the Diagnostic and Statistical Manual of Mental Disorders, include increased craving for or effort spent seeking alcohol, impaired control over alcohol intake, and personal and social impairments as a result of alcohol use (American Psychiatric Association, 2013). Understanding why some individuals develop AUD while others do not, and why some individuals decide to abstain entirely, holds significant clinical and societal importance.

Alcohol self‐administration is a complex, multifaceted decision‐making process. Understanding the behavioral processes that govern this behavior can yield important insights into the progression from recreational to pathological alcohol use. Preclinical models of alcohol self‐administration have delineated three major behavioral components: (1) the reinforcing effects of alcohol, (2) alcohol‐seeking (appetitive) behaviors, and (3) alcohol intake (consummatory) behaviors (for review, see Samson & Czachowski, 2003). Importantly, these components are dynamic and modifiable, rather than static.

For instance, the reinforcing value of alcohol depends not only on an individual's prior history and subjective experience with the drug, but also on the current context. This includes the organism's physiological state and interoceptive processing (for review see Lovelock et al., 2021), environmental cues (e.g., presence of alternative reinforcers, effort required to obtain alcohol, and sensory characteristics, like taste), and predictions about the potential consequences of drinking in that moment (Acuff et al., 2023; Acuff, Oddo, et al., 2024; Acuff, Strickland, et al., 2024). Human studies have provided compelling evidence for the role of contextual influences in alcohol‐related decision making (Acuff, Oddo, et al., 2024). While these studies have advanced our understanding of the nuanced and dynamic nature of alcohol use in real‐world settings, preclinical rodent models are uniquely positioned to dissect how specific, controlled manipulations affect each facet of the self‐administration process. These models also allow for mechanistic investigations of how risk factors (e.g., stress) and protective interventions (e.g., pharmacological treatments) influence reinforcement, seeking, and intake, along with their underlying neural circuitry.

The current review aims to (1) describe the behavioral processes involved with ethanol self‐administration, (2) detail preclinical approaches used to study these processes, and (3) review key advances that have emerged from one particularly well‐validated methodological approach, the biphasic sipper model of operant self‐administration. In light of recent developments in behavioral data analysis since the introduction of the biphasic sipper model, we also highlight how lickometer‐based operant paradigms can yield richer insights into self‐administration. To illustrate this potential, we present an open‐source analysis pipeline and demonstrate its utility using a compiled dataset of over 150 male Long Evans rats generated by our laboratory. Finally, we identify remaining gaps in the literature, with a focus on how preclinical methodologies can be further refined to advance our understanding of ethanol self‐administration. Importantly, accurately modeling the core behavioral and neurobiological features of AUD increases the likelihood of identifying promising therapeutic targets.

## BEHAVIORAL PROCESSES CONTROLLING ETHANOL SELF‐ADMINISTRATION

Ethanol self‐administration is interdependent on two behaviors: (1) it requires one to seek and ultimately obtain ethanol in order to (2) consume ethanol. In fact, appetitive (i.e., seeking) and consummatory behaviors are required for the organization of most behavior, not just ethanol self‐administration (Craig, 1918). Appetitive behaviors describe the degree to which an animal directs its behavior in order to procure ethanol (“How motivated are you to drink?”. “How frequently do you drink?”). These behaviors are shaped by learning and experience, as animals must understand the contingencies which result in ethanol procurement (Samson & Czachowski, 2003). In contrast, consummatory behaviors describe how much and how quickly ethanol is consumed once it is available. These unconditioned responses are critical for understanding intake control, a core feature disrupted in AUD.

While successful self‐administration requires both appetitive and consummatory behaviors, decades of research has demonstrated that appetitive and consummatory processes are differentially influenced by pharmacological and environmental manipulations (see Insights from the biphasic sipper model). Broadly, it is thought that changes in appetitive measures may model changes in wanting or craving while changes in consummatory measures may suggest alterations in postingestive pharmacological effects. While both are essential for modeling ethanol self‐administration, dissociating these processes is key to identifying how specific interventions affect motivation versus pharmacological reinforcement.

## PRECLINICAL MODELS TO STUDY ETHANOL CONSUMPTION

### Home cage drinking paradigms

Home cage drinking paradigms are among the most used preclinical models to study ethanol drinking due to their simplicity and high throughput. These models include forced and free‐choice exposure, delivered on continuous or intermittent schedules (Simms et al., 2008). While forced exposure can overcome neophobia and promote initial intake (Hiller‐Sturmhöfel & Kulkosky, 2001; Sardarian et al., 2020), it lacks face validity, as ethanol is the only available fluid. Free‐choice paradigms, such as the two‐bottle choice test, offer greater translational relevance by allowing voluntary ethanol intake and enabling preference measurements.

Although home cage paradigms are methodologically efficient, they offer limited resolution of behavioral processes. Traditional setups often lack precise data on drinking temporal dynamics and lick bout structure, making it difficult to distinguish between changes in seeking versus consumption. Moreover, voluntary intake in home cage models does not reliably predict operant self‐administration (Samson & Czachowski, 2003; Wheeler et al., 2025; Yoon et al., 2025), suggesting distinct underlying behavioral mechanisms. Recent advances, such as the integration of lickometer systems, are beginning to address these limitations by enabling more detailed analysis of drinking behavior with minimal experimenter interference (Godynyuk et al., 2019; Hou et al., 2024; Petersen et al., 2023; Yoon et al., 2025). Importantly, lickometer systems allow for the recording of the number and temporal dynamics of licking behaviors, resulting in the procurement of translationally relevant variables, such as lick rate and drinking microstructures. While these technological advances will certainly increase our understanding of intake behaviors under low cost and high availability conditions, home cage methods are unable to disentangle motivational aspects from consummatory processes.

### Operant models of self‐administration

Operant self‐administration procedures are widely used to assess appetitive behaviors alongside consummatory measures. In these paradigms, the completion of a behavioral response (e.g., lever press or nose poke) results in ethanol access, typically within an operant box that serves as a discrete self‐administration context. Ethanol access is determined by an experimenter‐selected schedule of reinforcement. Ratio schedules of reinforcement result in a consequence (i.e., access to ethanol) after a number of behavioral responses are completed, while interval schedules of reinforcement result in a consequence following a specific passage of time (Ferster & Skinner, 1957). Under these schedules of reinforcement, the number of reinforcers earned and/or the number of completed behavioral responses is commonly used as the appetitive dependent variable(s).

The most used schedule of reinforcement in operant models of self‐administration is fixed ratio (FR), resulting in a back‐and‐forth engagement with the operandum and the delivery method. Throughout most of the 20th century, the predominant delivery method was the “dipper” method, in which completion of a FR requirement resulted in the presentation of a small liquid volume via a discrete receptacle (typically 0.1–0.5 mL) (Samson, 1988). Practical limitations of this approach have been previously discussed (Blegen et al., 2018; Samson & Czachowski, 2003). For example, dipper‐style procedures assume that each earned reinforcer (i.e., FR completion) results in consistent and complete consumption of the available liquid. However, only some studies explicitly account for fluid loss due to spillage or evaporation.

To circumvent these limitations, sipper tube delivery methods have become more popular. One major advantage of sipper tubes is that they are closed units and therefore prevent evaporation during a given self‐administration session. Additionally, sipper tube delivery methods can be readily paired with lickometer systems, allowing for the quantification of licking behaviors at high temporal resolution to complement the measurement of fluid (mLs) consumed from the sipper tube. The inclusion of lickometer‐derived independent variables has allowed for a robust analysis of consummatory behaviors (discussed in greater detail in Standardized analysis of biphasic sipper model data). Like the dipper method, the sipper tube delivery method is also commonly paired with a FR schedule of reinforcement (Blegen et al., 2018; Patwell et al., 2021; Pitock et al., 2025; Yoon et al., 2025). In this delivery method, the completion of the FR results in fixed time access to the sipper tube (e.g., 30 s), after which the sipper tube is retracted and the FR can be completed again for subsequent access to the sipper tube.

FR schedules of reinforcement have been widely used to study self‐administration of various drug classes, such as stimulants administered intravenously (i.v.), where rapid absorption facilitates immediate pharmacological effects. For example, a single i.v. infusion of cocaine can elicit an increase in dopamine within 5 s (Espana et al., 2008). In contrast, oral ethanol self‐administration presents unique challenges due to first‐pass metabolism and variable absorption influenced by biological and environmental factors (Jones, 2019). These factors, combined with small‐volume delivery methods, can blunt achieved blood ethanol concentrations (BECs), due to BEC being sensitive to both the volume and speed of consumption (Jeanblanc et al., 2019; Samson & Czachowski, 2003). While FR schedules can be effective when paired with sufficient access duration and/or volume, short, limited‐access sessions may limit the ability of rodents to achieve meaningful pharmacological effects. Thus, selecting an appropriate sipper access duration is a key experimental decision that can substantially influence behavioral outcomes. If access is too brief, the limited intake may prevent rats from achieving pharmacologically meaningful BECs. Conversely, if access is too long, subsequent appetitive behaviors may be influenced by ethanol's rate‐altering effects (Hendler et al., 2011). Additionally, animals may reach saturation of their preferred dose early in the session, reducing subsequent lever pressing and limiting the assessment of motivation or seeking behavior.

These limitations highlight a potential divergence between rodent and human self‐administration. In humans, alcohol is typically consumed in larger quantities per drinking episode, often in a single sitting without enforced breaks. It is usually ingested for its pharmacological effects, which are closely tied to the volume and rate of consumption and often paired with more palatable taste components. In contrast, rodent models often assume that animals will expend effort to earn small, often bitter, volumes of ethanol that may not immediately produce reinforcing effects. Such discrepancies may be a key factor in the difficulty of translating preclinical discoveries into clinical interventions.

**FIGURE 1:**
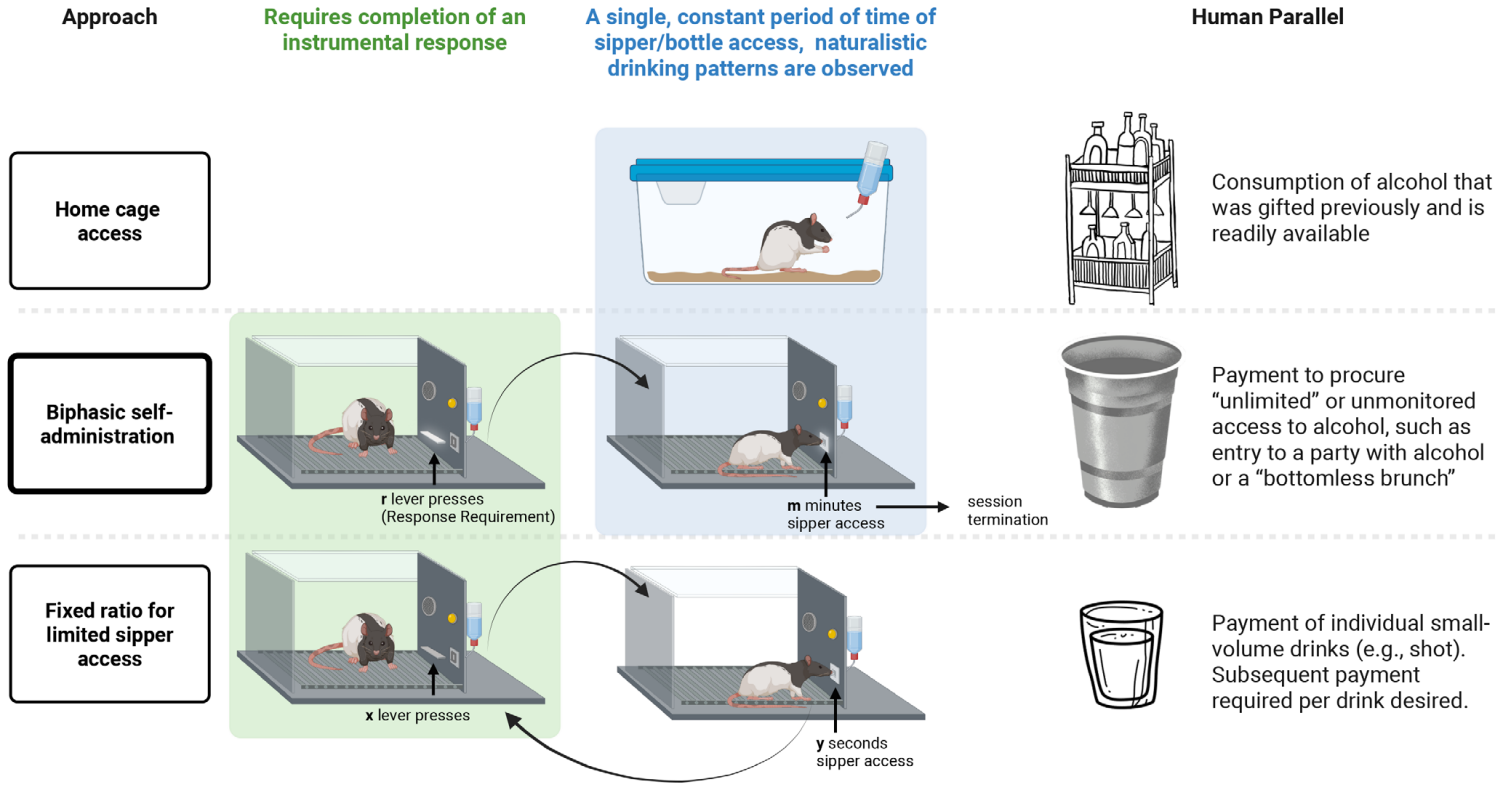
Schematics of three preclinical approaches to study ethanol consumption. Home cage access represents conditions where alcohol is freely available without instrumental requirements, paralleling human consumption of gifted or readily available alcohol. The biphasic sipper model of operant self‐administration requires completion of a single fixed ratio (FR) at the beginning of the session to gain uninterrupted sipper tube access for a limited access (e.g., 20 min), analogous to paying for unlimited or monitored alcohol access (e.g., parties or “bottomless” alcohol deals). FR for limited sipper access models require each FR completion to grant brief or small‐volume alcohol access (e.g., via dipper or short (5–30s) sipper duration), paralleling payment for individual small‐volume drinks (e.g., shots). Green shading indicates phases requiring instrumental responses, while blue shading represents periods of naturalistic drinking. Human parallels are noted on the right for conceptual comparison. Created on BioRender.com.

A further conceptual limitation of FR operant paradigms is the conflation of appetitive (seeking) and consummatory (drinking) processes. Because ethanol access is contingent on completing a behavioral requirement, it becomes difficult to determine whether a given manipulation, such as a pharmacological treatment or neural circuit intervention, affects motivation to seek ethanol, ethanol consumption, or both. To address these concerns, a biphasic self‐administration paradigm using a retractable, motorized sipper tube was created by Dr. Hank Samson's laboratory (Samson et al., 1998). Before any ethanol is ingested, animals are given 20 min to complete a response requirement. Completion of the response requirement triggers the extension of the retractable sipper tube that provides an opportunity for an additional 20 min of uninterrupted drinking. For example, a response requirement of 20 is functionally equivalent to a FR20 schedule, where 20 lever presses result in sipper access. This paradigm is distinct from other FR schedules because the rodent only has one opportunity to complete the FR within a fixed time window to obtain “unlimited” ethanol access. While the appetitive phase is usually 20 min, rats typically complete the response requirement within 2.5–4 min (Figure S1B). Additionally, while there is a finite amount of ethanol in the sipper tube (typically ~35–40 mLs), rodents do not terminate their drinking due to consuming all available fluid, as fluid is remaining in the sipper tube at the end of the session. Crucially, because this appetitive phase occurs before any ingestion of ethanol, the assessment of seeking behavior is not confounded by ethanol's dose‐dependent rate‐altering effects (Hendler et al., 2011). The biphasic nature of this model allows for a clear delineation between appetitive and consummatory phases because once the rodent reaches the response requirement, the sipper extends and simultaneously the lever retracts, no longer allowing the rodent to engage with the operandum. This design yields two major benefits when considering ethanol self‐administration: (1) appetitive behaviors can be assessed without any influence of ethanol's pharmacology and (2) the rodent can drink their preferred volume and rate, without experimenter‐induced interruption. Taken together, this methodology occupies a middle ground between home cage drinking paradigms, where consummatory behaviors are minimally disrupted, and traditional operant FR procedures, which require repeated instrumental responses to access experimenter‐determined volumes of ethanol (Figure 1).

## INSIGHTS FROM THE BIPHASIC SIPPER MODEL

The biphasic sipper model paradigm has enabled empirical assessment of whether appetitive and consummatory processes involved in ethanol self‐administration are functionally independent and/or differentially sensitive to experimental manipulations. Decades of research support these hypotheses (see Tables 1 and 2). Although other operant procedures, including traditional FR paradigms using dipper or sipper delivery methods, have successfully assessed sensitivity to reinforcer type (Slawecki et al., 1997), the influence of biological and environmental risk factors (Lopez & Becker, 2014; McNamara et al., 2025; Pitock et al., 2025), treatment effects (Gonzalez‐Marin et al., 2018), and neurobiological mechanisms (Hellard et al., 2019; Jeanblanc et al., 2023), the biphasic sipper model provides a complementary framework allowing for the independent quantification of seeking and intake behaviors, as ethanol consumption does not rely on continued instrumental responding once sipper access is earned. The following sections detail how the biphasic sipper model has been employed to dissect the distinct components of ethanol self‐administration. Each subsection highlights a specific manipulation or variable that has been instrumental in advancing our understanding of appetitive and consummatory processes, offering a nuanced understanding of how these behaviors are regulated.

**TABLE 1:** Review of studies examining systemic pharmacological treatment effects on ethanol or sucrose self‐administration as assessed in the biphasic sipper model.

Article	Rodent strain	Sex	FR	Task*	Solution	Treatment	Dose range (mg/kg)	Appetitive behaviors		Consummatory behaviors	
								Ethanol	Sucrose	Ethanol	Sucrose
Czachowski et al. (2001)	Long Evans rats	♂	30	Baseline sessions	10E or 3S	Acamprosate, acute treatment	50–200	—	—	↓	—
Czachowski & DeLory (2009)	Long Evans rats	♂	2	EPT	10E or 2S	Acamprosate, extended treatment	50–200	↓	—	↓	↓
						Chronic intermittent ethanol vapor exposure + Acamprosate, extended treatment	50–200	—	↓	—	—
Ford et al. (2007)	C57BL/6J mice	♂	8	Baseline sessions	10E	Allopregnanolone	50–400 ng; ICV	—		↑	
Czachowski, Legg, & Stansfield (2006); Czachowski, Prutzman, & DeLory (2006)	Long Evans rats	♂	30	EPT	10E or 2S	Baclofen	0.3–3.0	↓	↓	↑	↓
Windisch & Czachowski (2018)	Wistar rats	♂	10	EPT and Intake Assessment	10E or 2S	BINA	5.0–20.0	—	—	—	—
Windisch & Czachowski (2018)	Wistar rats	♂	10	EPT and Intake Assessment	10E or 2S	LY379268	0.3–2.0	↓	↓	—	↓
Ford et al. (2009)	C57BL/6J mice	♂	16	Baseline sessions	10E or 5S	Mecamylamine	2.0–8.0	↓	↓	↓	↓
Sharpe & Samson (2001)	Long Evans rats	♂	16	Baseline sessions	10E or 3S	Naloxone	0.3–1.0	—	—	↓	↓
Henderson‐Redmond & Czachowski (2014)	P rats, Long Evans rats	♂	10	EPT and Intake Assessment	10E or 2S	Naltrindole	2.5–10.0	P rats: ↓ Long Evans: −	P rats: ↓ Long Evans: ↓	P rats: ↓ Long Evans: −	P rats: − Long Evans: −
						Naltrexone	0.1–10.0	P rats: ↓ Long Evans: ↓	P rats: ↓ Long Evans: ↓	P rats: ↓ Long Evans: ↓	P rats: ↓ Long Evans: ↓
Czachowski & DeLory (2009)	Long Evans rats	♂	20	EPT	10E or 2S	Naltrexone	0.1–1.0	↓	—	↓	↓
						Chronic intermittent ethanol vapor exposure + Naltrexone	0.1–1.0	↓	↓	↓	↓
Thorsell et al. (2005)	Wistar rats	♂	20	Fixed Time	10E	Neuropeptide Y microinjection into the lateral ventricle	5–20 μ g/5 μ l, ICV	↓		↓	
Sharpe & Samson (2002)	Long Evans rats	♂	20	Baseline sessions	10E	Nicotine	0.35–7.0	↓		↓	
Verplaetse et al. (2012)	P rats	♂	20	EPT	10E or 2S	Prazosin	0.5–1.5	↓	↓	↓	↓
Verplaetse & Czachowski (2015)	P rats	♂	20	EPT and Intake Assessment	10E or 1S	Prazosin, Propranolol, Prazosin+Prop	0.125–1.0 5.0 0.125–1.0 + 5.0	Prazosin: ↓ Propranolol: ↓ Prazosin + Propranolol: ↓	Prazosin: ↓ Propranolol: ↓ Prazosin + Propranolol: ↓	Prazosin: ↓ Propranolol: ↓ Prazosin + Propranolol: ↓	Prazosin: ↓ Propranolol: ↓ Prazosin + Propranolol: ↓
						Prazosin, Propranolol, Prazosin+Prop	0.125–1.0 0.3 (sc) 0.125–1.0 + 0.3 (sc)	Prazosin: ↓ Naltrexone: ↓ Prazosin + Naltrexone: ↓	Prazosin: ↓ Naltrexone: − Prazosin + Naltrexone: −	Prazosin: ↓ Naltrexone: ↓ Prazosin + Naltrexone: ↓	Prazosin: ↓ Naltrexone: − Prazosin + Naltrexone: ↓
Czachowski et al. (2002)	Long Evans rats	♂	20 or 4	EPT	10E	Remoxipride	0.5–15.0	↓		—	
Freedland et al. (2001)	Long Evans rats	♂	16	Baseline sessions	10E or 3S	SR141716A	0.3–3.0	↓	—	↓	↓
McCane et al. (2018)	Wistar rats, P rats	♂ and ♀	10 and 20	EPT and Intake Assessment	10E or 1S	Tolcapone	3–30	♂ P rats: ↓ ♂ Wistars: ↓ ♀ P rats: − ♀ Wistars: −	♂ P rats: ↓ ♂ Wistars: ↓	♂ P rats: ↓ ♂ Wistars: − ♀ P rats: − ♀ Wistars: −	♂ P rats: ↓ ♂ Wistars: −
Henderson‐Redmond & Czachowski (2014)	P rats, Long Evans rats	♂	10	EPT and Intake Assessment	10E or 2S	U50,488H	2.5–10.0	P rats: ↓ Long Evans: ↓	P rats: ↓ Long Evans: ↓	P rats: ↓ Long Evans: ↓	P rats: ↓ Long Evans: ↓
Czachowski et al. (2018)	P rats	♂	20	EPT and Intake Assessment	10E or 1S	Varenicline	0.3–2.0	—	—	↓	—

*Note*: Treatments were administered intraperitoneally, unless otherwise specified. Symbols indicate whether any reported dose increased (↑) or decreased (↓) the associated behavior, or whether no reported doses resulted in any change of (−) the associated behavior. *Specific task details: *Baseline sessions*: The specified response requirement must be met in order for the sipper to be extended for a fixed time (typically 20 min). *EPT*: regardless of the specified response requirement, a session in which no number of lever presses results in procurement of the sipper tube and the total number of lever presses is recorded. *Intake assessment*: regardless of the specified response requirement, a session in which only 1 lever press is required for 20 min of access to the sipper tube and consummatory behavior is assessed.

Abbreviations: E, ethanol; EPT, extinction probe trial; HAD rats, high alcohol‐drinking rat line; ICV, intracerebroventricular injection; P rats, alcohol‐preferring rat line; S, sucrose; SC, subcutaneous injection.

**TABLE 2:** Review of studies examining brain‐region‐specific pharmacological treatment effects on ethanol or sucrose self‐administration as assessed in the biphasic sipper model.

Article	Rodent strain	Sex	FR	Task*	Solution	Compound	Subregion	Appetitive behaviors		Consummatory behaviors	
								Ethanol	Sucrose	Ethanol	Sucrose
**Amygdala**											
Butler et al. (2014)	Long Evans rats	♂	16	EPT	10E or 3S	BRL (β3‐adrenoceptor agonist)	Basolateral amygdala	↓	—	—	—
McCool et al. (2014)	Long Evans rats	♂	30	EPT	10E2S	Alpha‐m5HT	Basolateral/lateral amygdala	↓	—		
Bach et al. (2023)	Long Evans rats	♂	30	EPT	10E or 3S	Chemogenetic inhibition	Basolateral amygdala projection to ventral hippocampus	↓	↓	—	—
Henderson & Czachowski (2012)	Long Evans rats	♂	10	Intake Assessment	10E2S or 2S	Neuropeptide Y	Central amygdala	—	—	—	—
**Locus Coeruleus**											
Deal et al. (2020)	Long Evans rats	♂	30	EPT	10E	Tonic stimulation of norepinephrine activity		–		↑	
						Phasic stimulation of norepinephrine activity		↓		↓	
**Nucleus Accumbens (NAc)**											
Czachowski et al. (2001)	Long Evans rats	♂	10	EPT	10E	Raclopride	Predominantly Nac core	↓		↓	
Samson & Chappell (2004)	Long Evans rats	♂	30	EPT	10E	Raclopride	NAc core	↓		—	
Czachowski (2005)	Long Evans rats	♂	10 or 20	Intake Assessment or EPT	10E or 2S	CGS12066B	NAc core	↓	—	—	—
						8‐OH‐DPAT	NAc core	—	—	↓	−
Windisch & Czachowski (2018)	Wistar rats	♂	10	Intake Assessment and EPT	10E or 2S	LY379268	NAc core	↓		—	
**Ventral tegmental area (VTA)**											
Czachowski et al. (2012)	Long Evans rats	♂	10 or 20	Intake Assessment or EPT	10E or 2S	CNQX		↓	—	—	—
						SCH23390		—	—	—	—
						Tetrodotoxin		↓	↓	—	—
Budygin et al. (2020)	Long Evans rats	♂	30	EPT	10E	Tonic stimulation	VTA to NAc dopamine projection	↓		—	
						Phasic stimulation	VTA to NAc dopamine projection	↑		—	

*Note*: Symbols indicate whether the manipulation (any reported microinjection dose or optogenetic stimulation) increased (↑) or decreased (↓) the associated behavior, or whether no reported doses resulted in any change of (−) the associated behavior. *Specific task details: *EPT*: regardless of the specified response requirement, a session in which no number of lever presses results in procurement of the sipper tube and the total number of lever presses is recorded. *Intake assessment*: regardless of the specified response requirement, a session in which only 1 lever press is required for 20 min of access to the sipper tube and consummatory behavior is assessed.

Abbreviations: E, ethanol; EPT, extinction probe trial; S, sucrose.

### Dissociating appetitive and consummatory processes

A major advancement provided by the biphasic sipper model is the ability to study oral ethanol consumption in a way that requires effort to obtain ethanol, while allowing the subject to control the rate and amount consumed without interruption (Figure 1). Unlike paradigms that deliver ethanol in small, discrete volumes, the biphasic sipper model supports more naturalistic drinking patterns by minimizing schedule‐induced disruptions. Indeed, early studies comparing the consummatory phase of the biphasic sipper model with consummatory behaviors from FR studies using dipper (Samson et al., 2000, 2003) or sipper (Ford et al., 2007) delivery methods have demonstrated that the biphasic sipper model produces greater intake, faster intake, and shorter latencies to begin drinking. These observations underscore the importance of considering how schedule parameters influence intake behaviors when evaluating the translational elements of self‐administration paradigms. Together, these findings highlight how the biphasic sipper model contributes a distinct and complementary approach to studying ethanol self‐administration, particularly by enabling uninterrupted, self‐paced consumption following a discrete appetitive phase.

Beyond its utility in assessing consummatory behaviors, the biphasic sipper model has also been used to quantify motivation to seek ethanol without the influence of ethanol's rate‐altering effects, which could confound behavioral responses through stimulation or sedation (Hendler et al., 2011). Two primary approaches have been developed within this framework to isolate and measure appetitive processes. One approach employs an across‐session progressive ratio schedule of reinforcement to derive an index of motivation while maintaining the procedural separation between seeking and drinking (Czachowski & Samson, 1999). In this task, the response requirement to gain access to the sipper tube increases across successive sessions. The primary dependent variable is breakpoint, defined as the highest number of lever presses a rodent completes to earn access. This design allows for daily self‐administration sessions in which the animal controls the rate of ethanol intake, yields a reliable motivational metric, and demonstrates strong test–retest reliability after multiple determinations (Czachowski & Samson, 1999). An additional strength of the across‐session design is that any failure to meet the response requirement is not confounded by the sedative effects of ethanol or by satiety, since the animal has not yet consumed ethanol in that session. A notable limitation of this approach is the extended time required to determine a single breakpoint value per subject, often spanning several weeks. Additionally, the session in which an animal ultimately fails to meet the response requirement is not known in advance, which limits the feasibility of testing acute pharmacological or environmental manipulations.

To circumvent these limitations, the extinction probe trial (EPT) was developed to quantify a subject's motivation to procure ethanol and identifies this variable in a single session (Samson et al., 2003). During an EPT, rodents lever press for the entire duration of the appetitive phase (typically 20 min), yet do not receive ethanol as a consequence of lever pressing. Importantly, there are no cues to signify to the subject that the sipper tube will not extend into the chamber during that session. The total number of lever presses completed within the EPT is the dependent variable used to infer a subject's motivation to procure ethanol. There is high test–retest reliability when assessing lever pressing during the EPT, allowing for multiple assessments using this behavioral approach (Samson et al., 2001). Moreover, our laboratory recently demonstrated that the total number of lever presses completed during the EPT is significantly, positively correlated with breakpoints derived from the across‐session progressive ratio schedule (Ortelli & Weiner, 2024). A striking quality of the EPT that makes it unique compared with other measures of motivation, like breakpoint, is that it operationally separates appetitive and consummatory variables. Many studies have found no relationship between the average intake during the session before or after an EPT and responding during an EPT (Chappell & Weiner, 2008; Czachowski et al., 2003; Samson et al., 2001, 2003, 2004; Samson & Chappell, 2001). Because this measure of motivation can be captured in a single session, the EPT has been used to investigate how pharmacological treatments can mediate motivation to procure ethanol, as well as putative circuits governing motivation to procure ethanol, and are discussed below (Dissecting pharmacological treatment effects on appetitive and consummatory behaviors and Assessment of brain regions/circuit‐specific manipulations).

### Sensitivity to response cost (“response requirement”)

Initial studies using the biphasic sipper model first probed whether changing the response requirement would affect appetitive and/or consummatory variables. The seminal paper by Samson et al. (1998) was the first to demonstrate that as the response requirement increased, the percentage of rats who met that response requirement would decrease, at the group level. Notably, rats did not adjust their ethanol intake to compensate for the increased effort needed to gain access, suggesting a lack of compensatory drinking in response to greater cost.

These results were replicated in studies modifying this paradigm to implement the across‐session progressive ratio schedule (Czachowski et al., 2003; Czachowski & Samson, 1999; Ortelli & Weiner, 2024; Sharpe & Samson, 2003). Serendipitously, in one study Chappell and Weiner (2008) identified a subgroup of rodents (*n* = 6/23) who would not press more than 10–20 times to earn 20 min access to ethanol. Rather than excluding these rats from the study, the authors allowed these subjects to self‐select themselves to a lower response requirement group. These 6 rats were only trained to a response requirement of 8 while the remaining 17 rats were trained to a response requirement of 30. During EPTs, rats trained to the response requirement of 8 exhibited significantly lower levels of lever pressing than those trained to 30, suggesting reduced motivation to seek ethanol. Interestingly, average daily ethanol intake did not differ between the two groups, reinforcing the idea that appetitive and consummatory behaviors are dissociable within this paradigm.

Together, these findings underscore the value of using response requirement manipulations to reveal both group‐level trends and individual differences in ethanol self‐administration. The lack of compensatory drinking in response to increased effort, coupled with the dissociation between seeking and intake behaviors, suggests that motivation to obtain ethanol may be governed by distinct neural or behavioral processes from those regulating consumption in rodents. Moreover, the identification of subgroups with differing sensitivity to response cost highlights the utility of this paradigm for modeling individual variability in AUD behavioral risk factors, which may have translational relevance for understanding vulnerability and resilience in human populations. These results also point to context‐dependent decision making, where environmental constraints (e.g., response cost) influence motivation to seek ethanol without necessarily altering overall consumption.

### Sensitivity to reinforcer solution: Taste, pharmacology, and value

Studies using the biphasic sipper model demonstrate that rodents flexibly adjust their behaviors based on the sensory and pharmacological properties of the reinforcer. Comparisons between self‐administration behaviors for ethanol, sucrose‐sweetened ethanol, and sucrose reveal that both taste and pharmacology contribute to ethanol's reinforcing effects (Carrillo et al., 2008; Czachowski et al., 2003; Czachowski, Prutzman, & DeLory, 2006; Samson et al., 1999, 2000, 2002, 2003; Samson & Chappell, 2002; Sharpe & Samson, 2003), a seemingly obvious yet important detail as humans' perception of ethanol's reinforcing effects is also influenced by these reinforced and conditioned stimuli. Indeed, rodents will consume more sweetened ethanol than nonsweetened ethanol (Sharpe & Samson, 2003), highlighting the role of taste on ethanol self‐administration. Additionally, “free” access to ethanol, but not sucrose, immediately prior to the self‐administration session has been shown to reduce subsequent self‐administered intake in a dose‐dependent manner, indicating behavioral sensitivity to ethanol's pharmacological effects (Samson et al., 2002). These data are particularly compelling, as they suggest that rats self‐administering ethanol terminate their drinking not due to gastric fullness (as rats self‐administering sucrose consume a greater volume during the preload period), but rather regulate their intake to achieve a desired ethanol dose. These data also provide justification for the use of a self‐administration paradigm that includes a discrete seeking phase when no ethanol is on board. Specifically, ingestion of ethanol during the preload period (but not sucrose or water) decreased the proportion of rats who subsequently completed the response requirement. This may reflect rate‐decreasing effects or satiation, which in turn could have reduced subsequent motivation.

Within‐subject comparisons in this model have also revealed flexible, solution‐specific regulation of intake. When rodents were presented with different ethanol concentrations across sessions, they adjusted total volume consumed, particularly during the initial bout of drinking, to maintain a relatively stable ethanol dose, an effect not observed with sucrose (Samson et al., 1999, 2003; Sharpe & Samson, 2003). This dose‐regulating behavior suggests that ethanol self‐administration in the biphasic sipper model is not purely habitual. If lever pressing and drinking were governed by habitual processes, they would be less sensitive to outcome devaluation (Keramati et al., 2011). On the contrary, outcome devaluation studies, such as pairing ethanol administration with lithium chloride‐induced malaise, resulted in significant reductions in ethanol intake and EPT responding the following day (Samson et al., 2004), and therefore are consistent with a goal‐directed, value‐based decision‐making process. Together, these results highlight the utility of the biphasic sipper model for dissecting the nuanced, context‐dependent mechanisms that govern self‐administration, including the interplay between sensory cues, pharmacological feedback, and learned value. This paradigm allows researchers to parse how animals integrate multiple sources of information to regulate intake after completing an instrumental response, offering a powerful framework for modeling the complexity of human alcohol use behaviors.

### Influence of biological and environmental risk factors on self‐administration

The sipper model has also been used to investigate how biological and environmental risk factors may influence appetitive and consummatory processes. Many studies have investigated how alcohol‐preferring (“P”) and high alcohol‐drinking (HAD1 and HAD2) inbred rat lines behave in this self‐administration task (Beckwith & Czachowski, 2014; Bertholomey et al., 2013; Czachowski et al., 2018; Czachowski & Samson, 2002; Henderson‐Redmond & Czachowski, 2014; McCane et al., 2018; Verplaetse et al., 2012; Verplaetse & Czachowski, 2015). Studies have demonstrated that P rats have greater appetitive and consummatory behaviors than outbred lines, like Long Evans (Beckwith & Czachowski, 2014; Henderson‐Redmond & Czachowski, 2014) and Wistar (McCane et al., 2018) rats. Among all three alcohol‐preferring lines, P rats have demonstrated greater appetitive and consummatory behaviors than HAD1 and HAD2 rats, whereas the HAD lines have demonstrated comparable behavior to one another (Beckwith & Czachowski, 2014; Czachowski & Samson, 2002). These baseline differences highlight the need to consider genetic background when evaluating pharmacological interventions for AUD. Indeed, studies have reported differential effects on appetitive and consummatory behaviors when assessing potential pharmacological therapeutics between P and outbred rats (Henderson‐Redmond & Czachowski, 2014; McCane et al., 2018; Table 1).

In addition to genetic risk, developmental factors like early ethanol exposure can also shape later behavior in this model. Amodeo et al. (2017) reported that rats with a history of home cage ethanol exposure during adolescence had greater appetitive behavior when tested in adulthood compared with rats who did not have a history of ethanol exposure in adolescence. These preclinical findings are congruent with the human literature which demonstrates that adolescent alcohol use can have long‐lasting consequences, including greater risk of AUD development, which persist throughout adulthood (Spear, 2018). Additionally, McCool and Chappell (2009) reported that male Long Evans rats with a history of social isolation, a commonly used preclinical early life stress model, have increased lever press rates and ethanol intake compared with group‐housed control rats. These results may be caused by alterations in stress circuitry, a finding that complements the work of Bertholomey et al. (2013) who reported that yohimbine, a pharmacological stressor, increased reinstatement of ethanol seeking as well as ethanol intake in P and HAD2 male rats.

These findings demonstrate that the biphasic sipper model is well‐suited for probing how biological and environmental risk factors independently influence appetitive and consummatory components of self‐administration. The ability to detect baseline differences across genetic lines, as well as altered behavior following adolescent ethanol exposure or early life stress, underscores the model's sensitivity to individual and developmental variability. Moreover, the differential effects of pharmacological interventions across vulnerable populations highlight the importance of considering genetic and experiential background when evaluating potential treatments. Notably, despite the breadth of work examining strain, developmental, and stress‐related influences, relatively few studies (Haines et al., 2025; McCane et al., 2018; Ortelli & Weiner, 2024) have investigated sex differences using the biphasic sipper model. This represents a critical gap given the growing recognition of sex as a biological variable in addiction research. By integrating these risk factors into a self‐administration framework that separates seeking from consumption, the biphasic sipper model offers a powerful tool for identifying mechanisms underlying AUD vulnerability and for informing personalized therapeutic strategies.

### Dissecting pharmacological treatment effects on appetitive and consummatory behaviors

Following efforts to characterize and validate the biphasic sipper model as a preclinical model of oral ethanol self‐administration, many studies have assessed how various systemic treatments influence appetitive and consummatory behaviors (Table 1). A common finding across these studies is that systemic treatments frequently exert dissociable effects on appetitive versus consummatory behaviors (Czachowski et al., 2018; Czachowski et al., 2001; Czachowski et al., 2002; Czachowski, Legg, & Stansfield, 2006; Ford et al., 2007; Freedland et al., 2001; Henderson‐Redmond & Czachowski, 2014; McCane et al., 2018; Czachowski et al., 2018; Verplaetse et al., 2012; Windisch & Czachowski, 2018). The inclusion of sucrose controls has further allowed researchers to determine whether systemic treatment effects are specific to ethanol or reflect broader suppression of motivational behavior (Czachowski et al., 2001, 2018; Czachowski & DeLory, 2009; Czachowski, Legg, & Stansfield, 2006; Ford et al., 2009; Freedland et al., 2001; Henderson‐Redmond & Czachowski, 2014; McCane et al., 2018; Sharpe & Samson, 2001; Verplaetse et al., 2012; Verplaetse & Czachowski, 2015; Windisch & Czachowski, 2018).

Importantly, studies showing that FDA‐approved medications such as acamprosate (Czachowski et al., 2001) and naltrexone (Czachowski & DeLory, 2009; Henderson‐Redmond & Czachowski, 2014; Sharpe & Samson, 2001) produce predictable effects on self‐administration provide additional evidence for the model's construct validity. The ability to dissociate seeking from intake phases of self‐administration also allows for a more precise understanding of the behavioral specificity of these treatments (Czachowski et al., 2001) and novel compounds (Czachowski et al., 2018), as well as identifying the lack of behavioral specificity in others (Czachowski & DeLory, 2009; Henderson‐Redmond & Czachowski, 2014; Sharpe & Samson, 2001).

Strain differences are also apparent, with alcohol‐preferring (P) rats showing greater sensitivity to treatment effects than outbred strains (Henderson‐Redmond & Czachowski, 2014; McCane et al., 2018), and sex differences remain underexplored, with limited data available for female subjects (McCane et al., 2018). Taken together, these results highlight the potential of the biphasic sipper model to inform the development of more targeted and effective AUD treatments. However, the identification of a systemic treatment that is behaviorally specific and can reduce ethanol‐directed behaviors without reducing all reward‐seeking behaviors remains challenging. Indeed, despite promising preclinical findings, no new medications have been approved for AUD in over two decades. Integrating models like the biphasic sipper model with additional elements that address core diagnostic criteria for AUD, such as the inclusion of an alternative reinforcer to capture choice behaviors, may improve the alignment between preclinical efficacy and clinical outcomes.

### Assessment of brain regions/circuit‐specific manipulations

Further studies have used the biphasic sipper model to investigate how manipulations of specific brain regions implicated in addiction, such as the nucleus accumbens (NAc), ventral tegmental area (VTA), and amygdala, affect self‐administration (Table 2). Most studies have reported that microinjections of pharmacological agents, typically dopamine antagonists or serotonin agonists, into the NAc (Czachowski, 2005; Samson & Chappell, 2004; Windisch & Czachowski, 2018), VTA (Czachowski et al., 2012), and basolateral amygdala (Butler et al., 2014; McCool et al., 2014) reduced appetitive but not consummatory behaviors. This is particularly interesting given that accumbal dopamine responses have been observed during the consummatory phase (Doyon et al., 2005; Carrillo & Gonzales, 2011). These localized observations and interventions allow for greater precision than systemic treatments and support the idea that specific brain regions distinctly modulate different phases of ethanol self‐administration.

Even more precise approaches have leveraged modern neuroscience tools to dissect circuit‐specific contributions to behavior. For instance, Bach et al. (2023) used chemogenetics to inhibit projections from the basolateral amygdala to the ventral hippocampus and observed reduced EPT responding for both ethanol and sucrose, without altering intake. Similarly, Deal et al. (2020) employed optogenetics to show that tonic stimulation of locus coeruleus norepinephrine neurons increased ethanol intake without affecting seeking, while phasic stimulation decreased both seeking and intake. Budygin et al. (2020) further demonstrated that tonic versus phasic stimulation of VTA to NAc dopamine projections led to opposite effects on EPT responding by decreasing and increasing it, respectively, again without influencing intake. Together, these studies build upon the foundational behavioral work characterizing the biphasic sipper model, showing that distinct neural circuits govern appetitive and consummatory phases of ethanol‐related behavior.

One particularly interesting shared result among these findings is that, of the specific brain regions studied (primarily the NAc and amygdala), consummatory behaviors appear relatively resistant to change once sipper access is earned. The biphasic sipper model offers a distinct advantage in detecting this dissociation, as traditional FR paradigms might only reveal reductions in reinforcers earned, without distinguishing whether the manipulation affected seeking or consummatory processes. The current data suggest that region‐specific interventions may be particularly effective in modulating seeking behaviors. However, future studies should incorporate appropriate controls to rule out nonspecific rate‐altering effects and confirm the specificity of these behavioral changes, such as the inclusion of choice‐based experimental designs.

## STANDARDIZED ANALYSIS OF BIPHASIC SIPPER MODEL DATA

Recent advances in operant paradigms, particularly the growing use of lickometer‐equipped sipper devices, have generated increasingly rich, high‐resolution datasets. Despite this influx of detailed behavioral data, there remains a lack of standardization in how these data are processed and analyzed across laboratories (although see Brown et al., 2023). For researchers using lickometers, this lack of standardization can obscure key behavioral metrics, such as microstructural licking patterns or bout dynamics, which are increasingly recognized as translationally relevant.

### Analysis pipeline

To enhance our laboratory's analysis pipeline, the first author developed MedParser, an open‐source R package that easily extracts information from text files, such as those generated from Med Associates operant sessions (Ortelli & Colarusso, 2024). Additionally, the first author applied the utility of the functions from this package to create a Shiny application specific to the analysis of data generated from the biphasic sipper model (with a documentation site available at https://oortelli.github.io/MedParser). This application allows users to load raw data, define bout details, extract session‐level dependent variables (Table 3), visualize cumulative lever press and lick data, and easily export final datasets to Microsoft Excel for downstream analyses. The application is designed for broad accessibility: users without coding experience can use it via the Shiny interface, while advanced users may modify the source code to tailor it to specific experimental datasets and scientific questions.

**TABLE 3:** Description of variables generated to analyze self‐administration behavior from the sipper model.

Variable	Description
Latency to first LP	Cumulative time (s) elapsed from session start until the first lever press
Session start to last LP	Cumulative time (s) elapsed from session start until the last lever press
Time between first and last LP	Time (s) between the first and last lever press
LP bouts	Number of runs animal was pressing the lever without a 20 s pause between lever pressing *(threshold can be defined by user)*
Total LPs	Total number of lever presses within the session
LP rate: Session start to last LP	Number of lever presses per minute, time based on the difference between start of session and time of last lever press
LP rate: first to last LP	Number of lever presses per minute, time based on the difference between the time of the first and last lever presses
Latency to first lick	Cumulative time (s) elapsed from session start until first lick
Time between first and last lick	Time (s) between the first and last lick
Total lick time	Cumulative time (s) rat spends making contact with sipper, excluding time elapsed between drinking bouts
Lick bouts	Number of runs animal was making contact with sipper without a 20 s pause between contacts *(threshold can be defined by user)*
Lick bout nontrivial	Number of runs animal was making contact with sipper without a 20 s pause between contacts *and* number of events within this bout was greater than 50 *(thresholds can be defined by user)*
Total licks	Total number of licks
Lick rate: First to last lick	Number of licks per minute, time based on the difference between the first and last licks
Lick rate: Total lick time	Number of licks per minute, time based on total lick time which excludes the time elapsed between drinking bouts
First bout duration (%)	Percentage of duration (s) of first drinking bout compared with total time spent drinking
First bout lick (%)	Percentage of licks in first drinking bout compared with total number of licks

Abbreviation: LP, lever press.

The development of the Shiny application enabled extraction of behavioral variables that were previously difficult to quantify. Using this tool, we now generate seven key appetitive parameters (Figure S1) and ten key consummatory parameters (Figure S2) in our summary statistics (Table 3). One illustrative example of a nuanced variable made accessible by this approach is the distinction between the total time elapsed from the first to the last lick versus the actual time spent actively licking. Although this distinction may appear semantic, it yields significantly different values, suggesting that these variables capture distinct aspects of behavior (Figure S3).

### Illustrative application: Consummatory differences in ethanol‐ vs. sucrose‐drinking rats

Having generated these detailed behavioral variables, we sought to determine whether they could provide new insights into our understanding of self‐administration processes. To do so, we re‐analyzed 9 days of baseline self‐administration sessions from male Long Evans rats responding for 10% ethanol (*n* = 92) or 3% sucrose (*n* = 53). All behavioral data were collected by two female research staff members between 2016 and 2024. For each subject, consummatory and appetitive variables were averaged across completed sessions; sessions in which the response requirement was not met were excluded. However, due to variability in response requirements across cohorts (20, 25, or 30 lever presses), appetitive variables were not included in subsequent analyses to avoid confounding effects. Although prior studies have documented behavioral differences between ethanol and sucrose intake (Sensitivity to reinforcer solution: taste, pharmacology, and value), no previous work has examined these differences using such a comprehensive dataset. Therefore, our aim was to determine which consummatory variables most effectively distinguish ethanol‐ from sucrose‐consuming rats using unsupervised classification methods. To this end, we applied principal component analysis (PCA) and quadratic discriminant analysis (QDA) to session‐level consummatory data.

Before conducting PCA, we assessed Pearson correlations among variables (Table 1) to reduce redundancy. Lick bouts were removed due to high correlations (|r| > 0.80) with three other variables. Similarly, total lick time and percentage of licks completed during the first bout were excluded due to near‐perfect correlations with total licks and percentage of duration completed during the first bout, respectively. The remaining seven variables (Latency to First Lick, Time Between First and Last Lick, Lick Bouts Nontrivial, Licks, Lick Rate: First to Last Lick, Lick Rate: Total Lick Time, First Bout Duration Percentage) were centered and normalized prior to PCA, which was performed using the R (version 4.3.1) function prcomp(). A visual examination of the scree plot revealed an inflection point after the second principal component (PC), indicating that two components captured the majority of the variance while minimizing overfitting.

PC1 explained 49.99% of the variance and primarily reflected overall licking volume, with strong negative loadings for Licks, Lick Bouts Nontrivial, and Time Between First and Last Lick and a strong positive loading for First Bout Duration Percentage. These loadings covaried such that higher licking measures corresponded to lower PC1 scores, reflecting overall consummatory engagement defined by the magnitude and duration of sustained licking behavior. PC2 explained an additional 22.34% of the variance, representing differences in how rapidly rats engaged in licking once drinking began.

**FIGURE 2:**
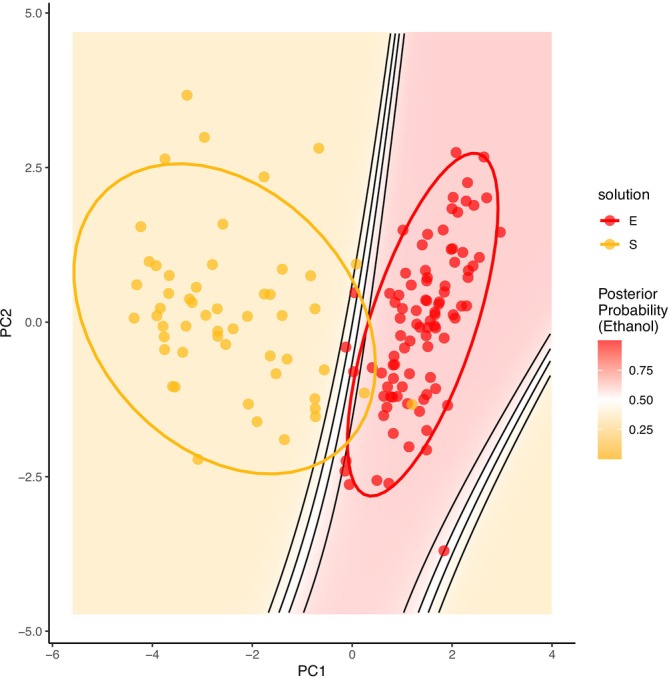
Quadratic discriminant analysis (QDA) decision boundaries and posterior probability contours for ethanol (E) and sucrose (S) groups in principal component analysis (PCA) space. Background shading represents posterior probability of ethanol classification (red = high, yellow = low), with black contour lines indicating probability thresholds (0.25, 0.50, and 0.75). The white background shading corresponds to a posterior probability of 0.5, where the model assigns equal likelihood to either group, indicating maximal classification uncertainty. Points represent individual subjects, colored by true group assignment (E = red, S = yellow), and ellipses denote 95% confidence regions for each group.

To explore whether the PCs captured meaningful group‐level structure, QDA was performed using the qda() function from the MASS package (Venables & Ripley, 2002). QDA was selected because Box's M test indicated that the covariance matrices differed significantly between the ethanol‐ and sucrose‐drinking subjects, violating the assumption of equal covariances (*χ* ^*2*^(55) = 740.7, *p* < 0.001). The model was trained on the first two principal components. Classification performance was evaluated using 10‐fold cross‐validation via the caret package (Kuhn, 2008), yielding an average accuracy of 96.6% (*κ* = 0.93). The receiver operating characteristic analysis indicated excellent discriminative ability of the QDA model (AUC = 0.99, 95% CI [0.97, 1.00]), consistent with the high cross‐validated accuracy and κ value. The final model trained on the full dataset demonstrated high sensitivity (97.8%) and specificity (96.2%), suggesting that the model reliably distinguished between ethanol‐ and sucrose‐drinking subjects (Figure 2).

**FIGURE 3:**
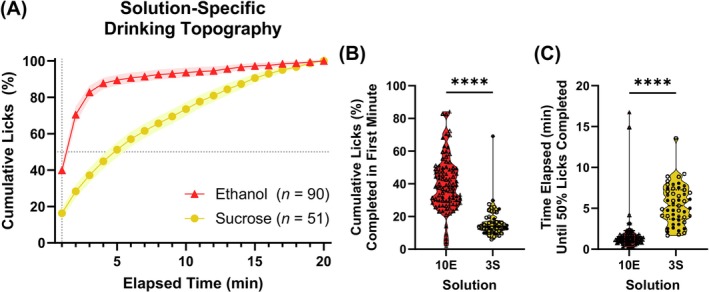
Cumulative lick curve data among the subjects correctly classified from the quadratic discriminant analysis (QDA). (A) Cumulative lick curve data from a representative baseline session were averaged among correctly classified subjects self‐administering ethanol (*n* = 90/92) or sucrose (*n* = 51/53) (mean with 95% Confidence Interval shading). Subjects self‐administering 10% ethanol (10E) and 3% sucrose (3S) demonstrate significant differences in (B) the percentage of cumulative licks completed during the first minute of sipper access and (C) the time at which half of all drinking was completed. Mann–Whitney tests were used to compare distributions of ethanol‐ and sucrose‐drinking rats. ****p < 0.0001.

To further characterize consummatory behavior, we analyzed cumulative lick patterns obtained in our application from the final baseline session, focusing on subjects correctly classified by the QDA. Unlike the session‐level variables used in PCA, cumulative lick curves offer a temporally resolved view of intake, capturing *how* subjects drink rather than simply *how much*. These curves normalize for total licks and reveal distinct patterns between ethanol and sucrose groups (Figure 3A). Mann–Whitney tests confirmed significant differences: ethanol rats completed a greater percentage of licks in the first minute (median = 38.22%) compared with sucrose rats (median = 14.16%; *U* = 376, *p* < 0.0001), and reached 50% of total licks more quickly (median = 1.3 min vs. 5.3 min; *U* = 188.5, *p* < 0.0001) (Figure 3B–C). Taken together, these data reinforce the well‐documented phenomenon of front‐loading in ethanol consumption (see Ardinger et al., 2022 for review), and demonstrate how cumulative lick curves and derived parameters can reveal dynamic intake patterns that are not evident from aggregate measures. In contrast to ethanol, sucrose consumption tends to be more evenly distributed across the session, reflecting a steady sipping pattern. Importantly, we have also observed these behavioral patterns in a within‐session ethanol and sucrose concurrent choice paradigm, further supporting that the solutions themselves, rather than prior drinking history, are driving these distinct consummatory patterns (Ortelli & Weiner, 2025).

### Additional applications of analysis pipeline

While the current data demonstrate the ability to use this code to evaluate consummatory data, this level of analysis can also be completed for appetitive variables. For example, our team recently used an earlier version of this program to demonstrate that inhibiting the projection from the basolateral amygdala to the ventral hippocampus altered lever pressing patterns such that activity in the first 5 min of the EPT, specifically, was especially blunted and never rebounded throughout the duration of the trial (Bach et al., 2023). Beyond analyzing data from the biphasic sipper model paradigm, this code was recently adapted to analyze bout‐level behavior in an i.v. self‐administration study (Dawes et al., 2024), demonstrating its flexibility across addiction research paradigms. Importantly, the pipeline presented is program‐agnostic and does not require Med Associates equipment, making it broadly accessible. Taken together, these analytical approaches offer a powerful and adaptable framework for examining behavioral data, enabling more precise assessments of both consummatory and appetitive behaviors across diverse models of self‐administration. As open‐source tools continue to be shared across laboratories, they hold significant potential to enhance the rigor and reproducibility of behavioral neuroscience research.

## CONCLUSIONS AND FUTURE DIRECTIONS

In this review, we examined the literature on appetitive and consummatory behaviors in operant ethanol self‐administration studies using the biphasic sipper model. We also introduced a new open‐source analysis tool to quantify these behaviors at higher resolution. Over the past 25 years, our understanding of the environmental and contextual influences, as well as the underlying neurobiological mechanisms, of ethanol self‐administration has advanced considerably. However, no new FDA‐approved medications for AUD have emerged during this time. These realities underscore the continued need for translationally relevant preclinical models to support the development of more effective AUD treatments. Our analytic pipeline contributes to the scientific effort to promote standardized, transparent, and reproducible analysis of high‐resolution behavioral data. While simpler analyses (e.g., total intake or session averages) can offer broad insights into self‐administration behavior, they can also obscure meaningful temporal and structural patterns in the data. The analytical pipeline described here enables a more nuanced characterization of both appetitive and consummatory behaviors, revealing dynamics such as front‐loading, bout structure, and rate of engagement that are not easily captured by aggregate measures. These features are particularly relevant in alcohol research, where subtle shifts in drinking patterns can reflect treatment effects.

While the biphasic sipper model and the presented accompanying analysis pipeline offer powerful means to quantify nuanced behaviors, important gaps remain. One notable gap in the literature is the limited investigation into how sex as a biological variable influences appetitive and consummatory phases of ethanol self‐administration, and whether the underlying neural circuits are sexually dimorphic. The biphasic sipper model is particularly well‐suited for investigating sex differences, as it allows subjects 20 min of free access to drink at their own pace. This design avoids a key confound present in fixed‐volume reinforcement schedules, where administering identical volumes (e.g., 0.1 mL per reinforcer) results in different ethanol doses (g/kg) across sexes due to differences in body weight. Female rats, for instance, require less volume to reach the same g/kg dose as males and would therefore have to lever press less, potentially conflating appetitive and consummatory behaviors.

To date, only three studies have employed both male and female subjects within the biphasic sipper model framework (Haines et al., 2025; McCane et al., 2018; Ortelli & Weiner, 2024). Our laboratory's recent work revealed no sex differences in appetitive responding despite significant differences in intake, a finding likely only possible due to the model's procedural dissociation of appetitive and consummatory components (Ortelli & Weiner, 2024). McCane et al. (2018) further demonstrated that a candidate pharmacological therapeutic reduced ethanol intake in male but not female P rats, reinforcing the importance of evaluating sex‐specific treatment effects. Notably, no studies to date have examined how sex chromosome complement or hormonal mechanisms influence these behaviors, an important future direction that could deepen our understanding of sex as a complex, multidimensional biological construct rather than a binary category (see Grissom et al., 2024).

Another key limitation in current preclinical operant designs is the near‐universal use of a single reinforcer, typically either ethanol or sucrose. In these designs, subjects are typically only exposed to one reinforcer, and any comparisons between reinforcers are made across groups rather than within‐subjects. This design limits ecological and translational validity, as humans typically have access to alcohol alongside alternative, nonalcohol reinforcers in real‐world contexts. Importantly, prioritizing alcohol over alternative reinforcers is a core diagnostic criterion for AUD and other substance use disorders (Banks & Negus, 2017). Future studies, especially those evaluating candidate therapeutics, would benefit from incorporating choice paradigms into operant models to more closely model human decision making. Our laboratory has recently developed an operant choice paradigm that leverages the benefits of the biphasic sipper model that we believe will be critical in advancing our understanding of the development, maintenance, and modulation of ethanol self‐administration (Ortelli & Weiner, 2025). We demonstrate that concurrent ethanol and sucrose availability decreases self‐administration of both solutions, particularly ethanol self‐administration, and that a manipulation can have solution‐specific effects within a single session. This framework could be extended even further to study concurrent self‐administration of multiple psychoactive substances, thereby addressing the rising prevalence of polysubstance use, a critical and emerging area of addiction science.

Together, the biphasic sipper model and associated analytical tools offer a robust framework for dissecting the behavioral components of ethanol self‐administration with greater precision and reproducibility. As the field continues to evolve, integrating these methodological advances with more nuanced experimental designs will be essential for bridging the gap between preclinical research and the development of effective, individualized treatments for AUD.

## CONFLICT OF INTEREST STATEMENT

The authors declare that they do not have any conflicts of interest (financial or otherwise) related to the data presented in this manuscript.

## Supporting information


Figures S1‐S3

## Data Availability

The data that support the findings of this study are openly available in GitHub at https://github.com/oortelli/MedParser.
