# Supplementary material for: Procedural separation of appetitive and consummatory behaviors in operant ethanol self‐administration: A review and open‐source analytical framework
Source: Alcohol Clin Exp Res (Hoboken). 2026 Feb 3;50(2):e70237. doi: 10.1111/acer.70237 (PMC12865747; doi:10.1111/acer.70237)
Supplement: Supplementary file 1 — Figures S1‐S3 [file ACER-50-0-s001.zip › acer70237-sup-0001-Supinfo1@S1_AppetitiveDescriptiveStats.pdf]

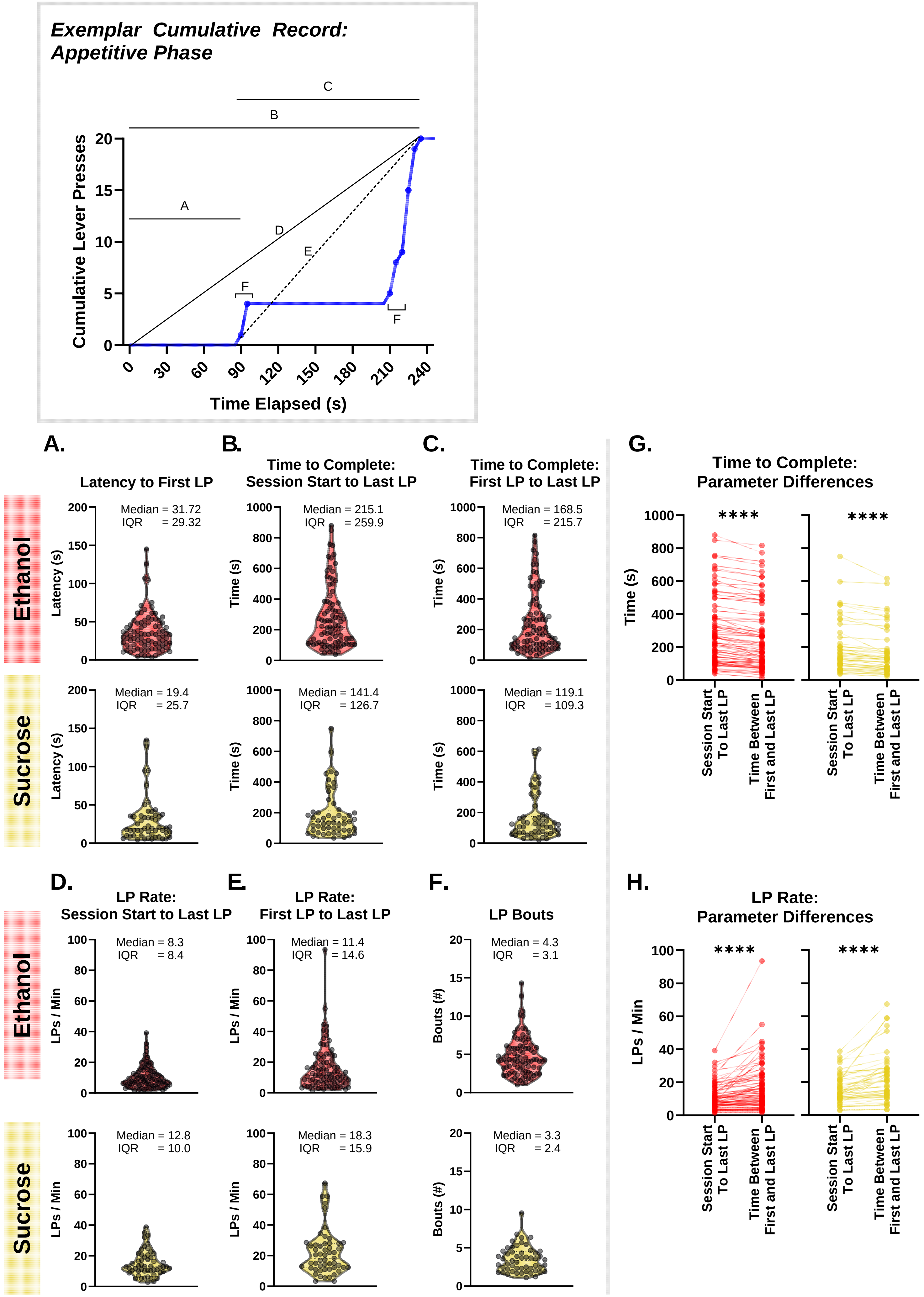

Exemplar Cumulative Record: Appetitive Phase
Cumulative Lever Presses
Time Elapsed (s)
A.
Latency to First LP
B.
Time to Complete: Session Start to Last LP
C.
Time to Complete: First LP to Last LP
G.
Time to Complete: Parameter Differences
Ethanol
Sucrose
Median = 31.72
IQR = 29.32
Median = 215.1
IQR = 259.9
Median = 168.5
IQR = 215.7
Median = 19.4
IQR = 25.7
Median = 141.4
IQR = 126.7
Median = 119.1
IQR = 109.3
Latency (s)
Time (s)
****
Session Start To Last LP
Time Between First and Last LP
D.
LP Rate: Session Start to Last LP
E.
LP Rate: First LP to Last LP
F.
LP Bouts
H.
LP Rate: Parameter Differences
Median = 8.3
IQR = 8.4
Median = 11.4
IQR = 14.6
Median = 4.3
IQR = 3.1
Median = 12.8
IQR = 10.0
Median = 18.3
IQR = 15.9
Median = 3.3
IQR = 2.4
LPs / Min
Bouts (#)
