# Supplementary material for: Procedural separation of appetitive and consummatory behaviors in operant ethanol self‐administration: A review and open‐source analytical framework
Source: Alcohol Clin Exp Res (Hoboken). 2026 Feb 3;50(2):e70237. doi: 10.1111/acer.70237 (PMC12865747; doi:10.1111/acer.70237)
Supplement: Supplementary file 1 — Figures S1‐S3 [file ACER-50-0-s001.zip › acer70237-sup-0002-Supinfo2@S2_ConsummatoryDescriptiveStats.pdf]

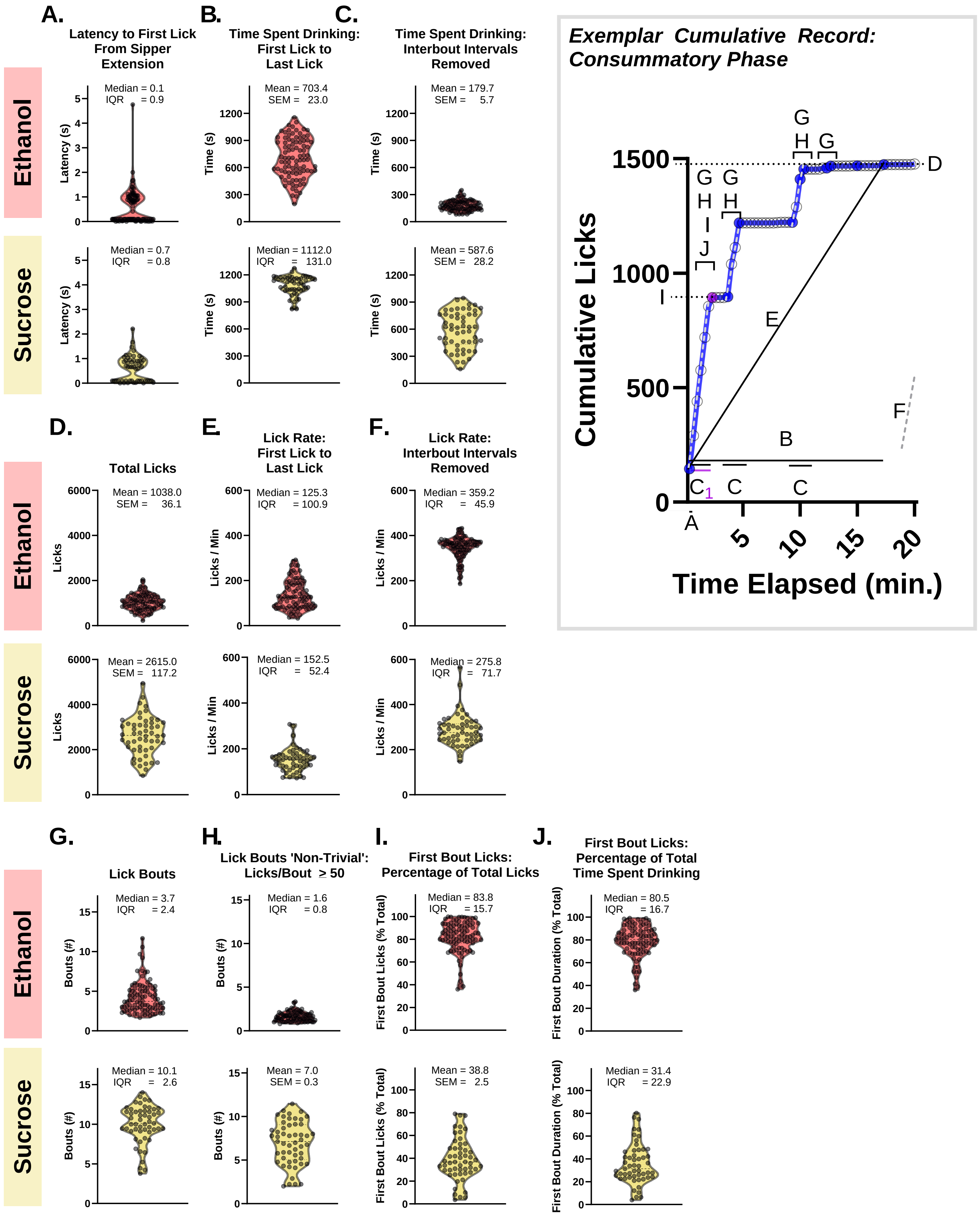

A.
Latency to First Lick From Sipper Extension
B.
Time Spent Drinking: First Lick to Last Lick
C.
Time Spent Drinking: Interbout Intervals Removed
Ethanol
Sucrose
Median = 0.1
IQR = 0.9
Mean = 703.4
SEM = 23.0
Mean = 179.7
SEM = 5.7
Median = 0.7
IQR = 0.8
Median = 1112.0
IQR = 131.0
Mean = 587.6
SEM = 28.2
Latency (s)
Time (s)
Exemplar Cumulative Record: Consummatory Phase
Cumulative Licks
Time Elapsed (min.)
D.
Total Licks
E.
Lick Rate: First Lick to Last Lick
F.
Lick Rate: Interbout Intervals Removed
Mean = 1038.0
SEM = 36.1
Median = 125.3
IQR = 100.9
Median = 359.2
IQR = 45.9
Mean = 2615.0
SEM = 117.2
Median = 152.5
IQR = 52.4
Median = 275.8
IQR = 71.7
Licks
Licks / Min
G.
Lick Bouts
H.
Lick Bouts 'Non-Trivial': Licks/Bout ≥ 50
I.
First Bout Licks: Percentage of Total Licks
J.
First Bout Licks: Percentage of Total Time Spent Drinking
Median = 3.7
IQR = 2.4
Median = 1.6
IQR = 0.8
Median = 83.8
IQR = 15.7
Median = 80.5
IQR = 16.7
Median = 10.1
IQR = 2.6
Mean = 7.0
SEM = 0.3
Mean = 38.8
SEM = 2.5
Median = 31.4
IQR = 22.9
Bouts (#)
First Bout Licks (% Total)
First Bout Duration (% Total)
