# Supplementary material for: Procedural separation of appetitive and consummatory behaviors in operant ethanol self‐administration: A review and open‐source analytical framework
Source: Alcohol Clin Exp Res (Hoboken). 2026 Feb 3;50(2):e70237. doi: 10.1111/acer.70237 (PMC12865747; doi:10.1111/acer.70237)
Supplement: Supplementary file 1 — Figures S1‐S3 [file ACER-50-0-s001.zip › acer70237-sup-0003-Supinfo3@S3_ConsummatoryParametricDiffs.pdf]

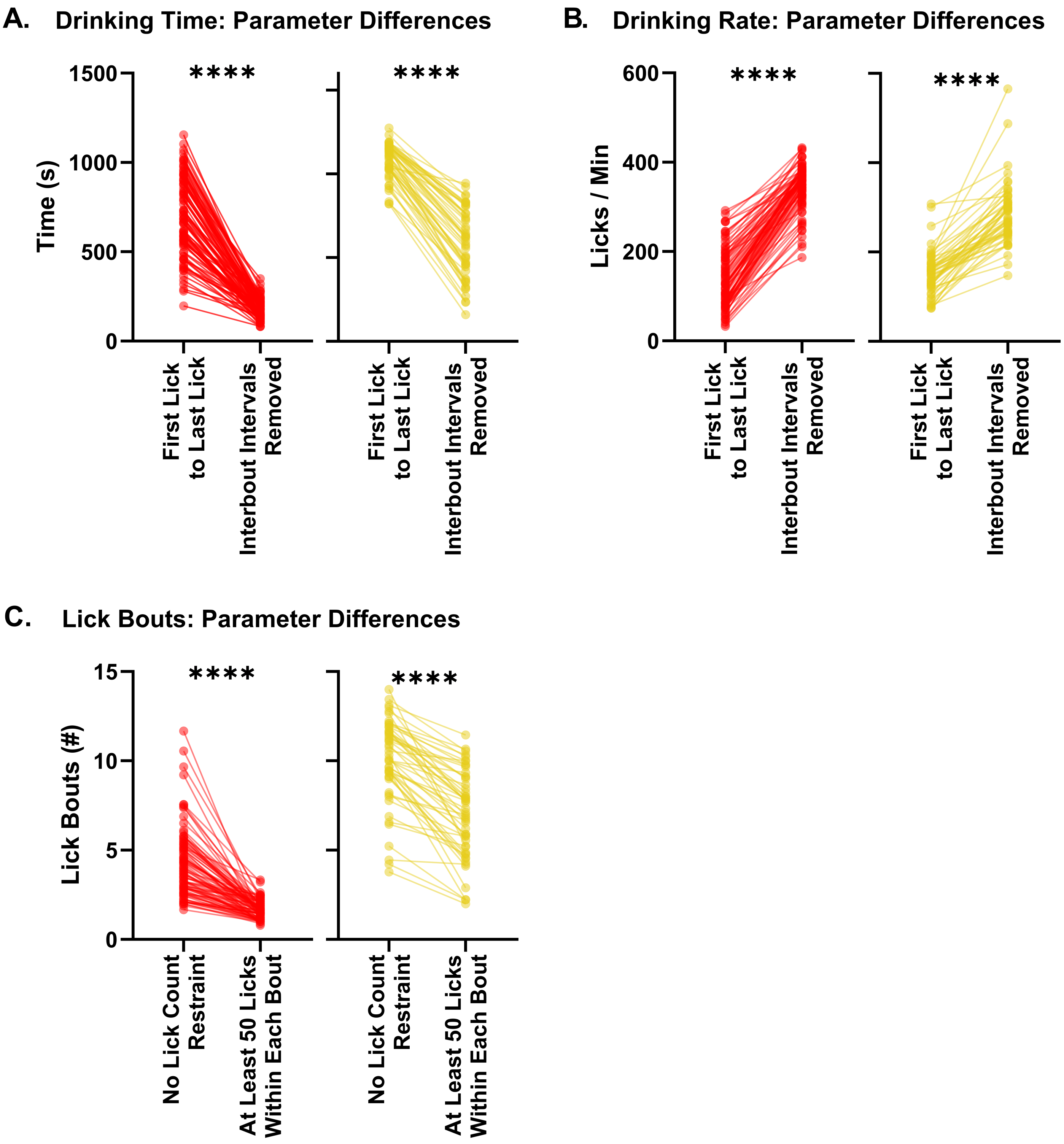
A.
Drinking Time: Parameter Differences
Time (s)
1500
1000
500
0
****
****
First Lick to Last Lick
Interbout Intervals Removed
B.
Drinking Rate: Parameter Differences
Licks / Min
600
400
200
0
****
****
First Lick to Last Lick
Interbout Intervals Removed
C.
Lick Bouts: Parameter Differences
Lick Bouts (#)
15
10
5
0
****
****
No Lick Count Restraint
At Least 50 Licks Within Each Bout
