# Supplementary material for: Procedural separation of appetitive and consummatory behaviors in operant ethanol self‐administration: A review and open‐source analytical framework
Source: Alcohol Clin Exp Res (Hoboken). 2026 Feb 3;50(2):e70237. doi: 10.1111/acer.70237 (PMC12865747; doi:10.1111/acer.70237)
Supplement: Supplementary file 1 — Figures S1‐S3 [file ACER-50-0-s001.zip › acer70237-sup-0004-Supinfo4@SupplementalFigureCaptions.docx]

**Supplemental Figure 1.**

Exemplar cumulative lever press (LP) record during the appetitive phase of the biphasic sipper model. Letters (A-F) on the cumulative record correspond to the associated behavioral parameters shown in panels A-F. In this example, the rat was required to complete 20 lever presses, after which the lever was retracted for the remainder of the session. Violin plots illustrate the distribution of behavioral variables in a sample of rats self-administering either 10% ethanol (*n* = 92) or 3% sucrose (*n* = 53). Because all variables are non-normally distributed, medians and interquartile ranges (IQR = 75th percentile – 25th percentile) are reported. Latency to first lever press (A) is defined as the time elapsed (in seconds) from session start to the first lever press. Time to complete the lever press requirement is assessed either as the time from session start to the last lever press (B) or the time between the first and last lever press (C). Welch’s paired t-tests revealed significant differences in both ethanol and sucrose groups when comparing these parameters (G), suggesting that they capture distinct behavioral phenotypes. LP rate is calculated as the number of presses per minute, using either the time from session start to last press (D) or the time between first and last press (E). Welch’s paired t-tests again revealed significant differences in both groups (H), supporting the interpretation that these measures reflect different behavioral strategies. Lever press bouts (F) are quantified as the number of discrete LP periods, defined such that no more than 20 seconds elapse between consecutive presses. *****p* < .0001

**Supplemental Figure 2.**

Exemplar cumulative lick record during the consummatory phase of the biphasic sipper model. Letters (A-J) on the cumulative record correspond to the associated behavioral parameters shown in panels A-J. In this example, the rat earned 20 minutes of access to a sipper tube. Violin plots illustrate the distribution of behavioral variables in a sample of rats self-administering either 10% ethanol (*n* = 92) or 3% sucrose (*n* = 53). Medians and interquartile ranges (IQR = 75th percentile – 25th percentile) are reported when variables are non-normally distributed while means and standard error of the mean are reported when variables are normally distributed. Latency to first lick (A) is defined as the time elapsed (in seconds) from sipper access to the first lick. Time spent drinking is assessed either as the time between the first and last lick (B) or the cumulative time contact is made with the sipper tube, therefore excluding interbout intervals (C). The total number of licks achieved in the 20 minute session is also recorded (D). Lick rate is calculated as the number of licks per minute, using either the time between first and last lick (E) or the time spent drinking with interbout intervals removed (F). Lick bouts (G) are quantified as the number of licking periods, defined such that no more than 20 seconds elapse between consecutive licks. We also record “non-trivial” lick bouts, such that a period is only counted if it also meets the condition that at least 50 licks occurred (H). To further quantify front-loading behavior, we also calculate the number of licks that occur during the first bout, relative to all licks achieved in the session (I) and the time spent drinking in the first bout, relative to total time spent drinking (interbout intervals removed) (J).

**Supplemental Figure 3.**

Welch’s paired *t*-tests were used to assess whether consummatory variables captured distinct behavioral phenotypes among male rats self-administering either 10% ethanol (*n* = 92) or 3% sucrose (*n* = 53). We observed a significant difference in parameter values when comparing the two time spent drinking parameters (A), the associated lick rate variables (B), and the lick bout variables without and with a 50 licks/bout constraint (C). *****p* < .0001.
